# Gene amplification in mesenchymal stem cells and during differentiation towards adipocytes or osteoblasts

**DOI:** 10.18632/oncotarget.22804

**Published:** 2017-12-01

**Authors:** Nora Corinna Altmayer, Valentina Galata, Nadine Warschburger, Andreas Keller, Eckart Meese, Ulrike Fischer

**Affiliations:** ^1^ Department of Human Genetics, Saarland University, 66421 Homburg/Saar, Germany; ^2^ Chair of Clinical Bioinformatics, Saarland University, 66123 Saarbrücken, Germany

**Keywords:** gene amplification, CDK4, MDM2, osteoblast, adipocyte

## Abstract

Gene amplifications are an attribute of tumor cells and have for long time been overlooked in normal cells. A growing number of investigations describe gene amplifications in normal mammalian cells during development and differentiation. Possibly, tumor cells have rescued the gene amplification mechanism as a physiological attribute of stem cells. Here, we investigated human mesenchymal stem cells (hMSCs) for gene amplification using array-CGH, single cell fluorescence *in situ* hybridization and qPCR. Gene amplifications were detected in mesenchymal stem cells and in mesenchymal stem cells during differentiation towards adipocytes and osteoblasts. Undifferentiated hMSCs harbor 12 amplified chromosomal regions, hMSCs that differentiated towards adipocytes 18 amplified chromosome regions, and hMSCs that differentiate towards osteoblasts 19 amplified regions. Specifically, hMSCs that differentiated towards adipocytes or osteoblasts harbor *CDK4* and *MDM2* amplifications both of which frequently occur in osteosarcoma and liposarcoma that are both of same cell origin. Beside the amplifications, we identified 36 under-replicated regions in undifferentiated and in differentiating hMSC cells.

## INTRODUCTION

Gene amplifications can be found in tumor cells, drug resistant cells and in cells essential for developmental processes in amphibians and flies [1]. Previously we reported gene amplifications during differentiation in human and mouse neural stem and progenitor cells [2–4] as well as in human and mouse myoblast cells during differentiation towards muscle cells [5]. In addition *ERBB2* gene amplification was reported in human trophoblast cells during differentiation [6] and amplification of placental genes in mouse giant trophoblast cells during differentiation [7]. These results leave the question to what extend gene amplifications during differentiation were a common attribute and if one can find gene amplifications in different progenitor cells including adipogenic progenitor cells or osteogenic progenitor cells during their differentiation towards adipocytes and osteoblasts.

Here we set out to analyze gene amplification in human mesenchymal stem cells and in human mesenchymal stem cell differentiation induced towards adipocytes and osteoblasts. Amplification analysis was performed using three techniques: using array CGH we determined amplified chromosomal regions, using fluorescence *in situ* hybridization (FISH) we confirmed gene amplifications on single cell level and using qPCR we verified the results with an independent method.

As previous results demonstrated an overlap of gene amplifications between differentiating cells and tumors derived from the same lineage, we investigated genes *CDK4* and *MDM2* for amplification because *CDK4* and *MDM2* amplifications were frequently reported in tumor cells e.g. in liposarcoma and in osteosarcoma [8, 9].

## RESULTS

### Differentiation towards adipocytes and osteoblasts

**Figure 1:**
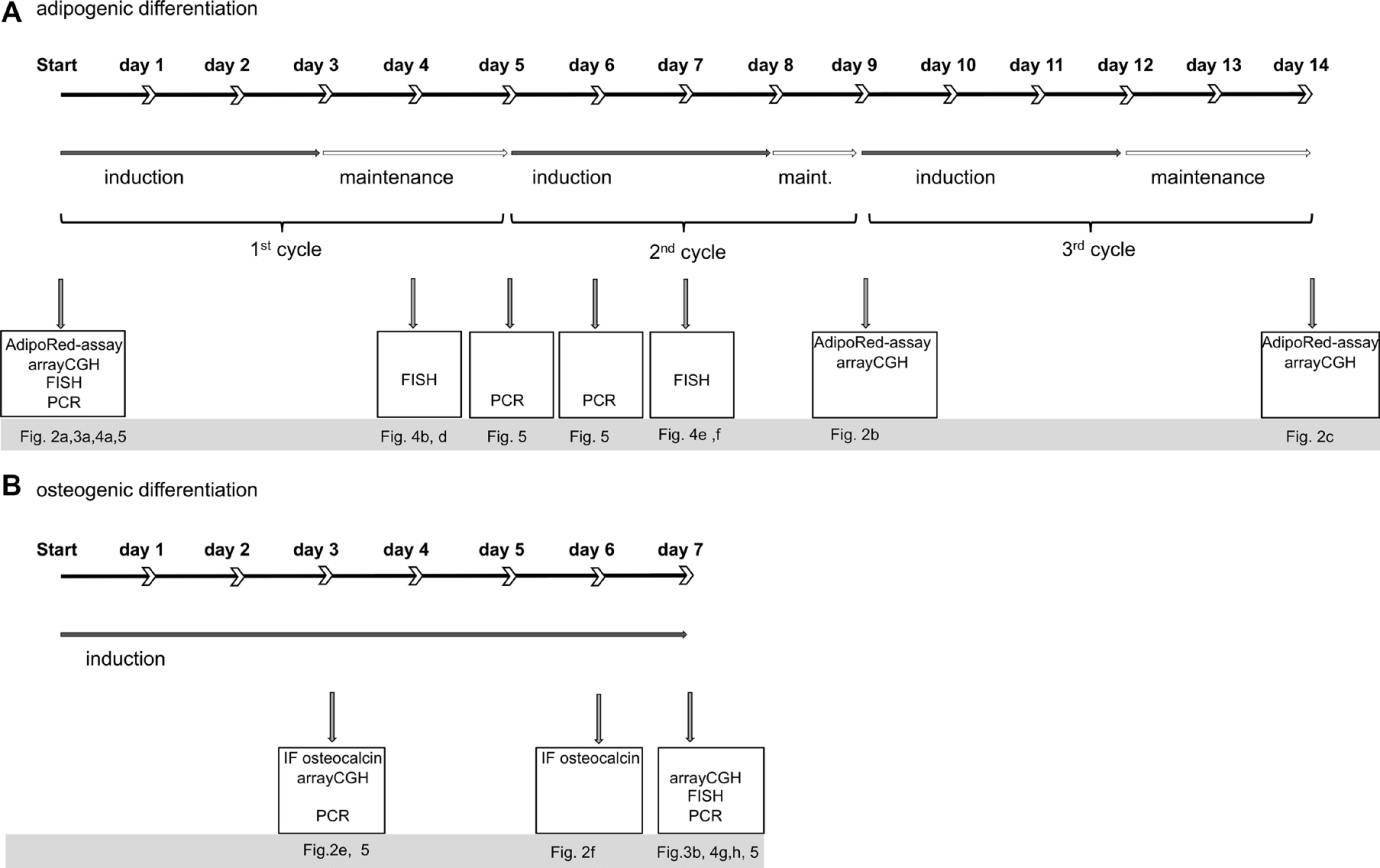
Overview on differentiation scheme and methodology (**A**) graphical time scale is displayed for adipogenic (a) and osteogenic (**B**) differentiation of human mesenchymal stem cells. Each cycle of adipogenic induction is divided in culture with induction medium (closed arrow) and maintenance medium (open arrow). Boxes display experiments performed at the indicated time points and corresponding Figures.

Human mesenchymal stem cells (hMSC) that were obtained from Lonza in passage 2 were cultured in mesenchymal stem cell maintenance medium, passaged once and further cultured until cells reached 80–90% density. All differentiation experiments were finished within 22 days after initial seeding. To obtain an overview on the occurrence of amplifications prior and after differentiation induction, we analyzed different time points by different techniques. Besides arrayCGH, we applied FISH and qPCR both of which allow detecting amplifications in small subpopulations of cells. FISH and qPCR were specifically used to identify gene amplification early in differentiation. An overview on the differentiation time scale and applied techniques is shown in Figure 1.

**Figure 2:**
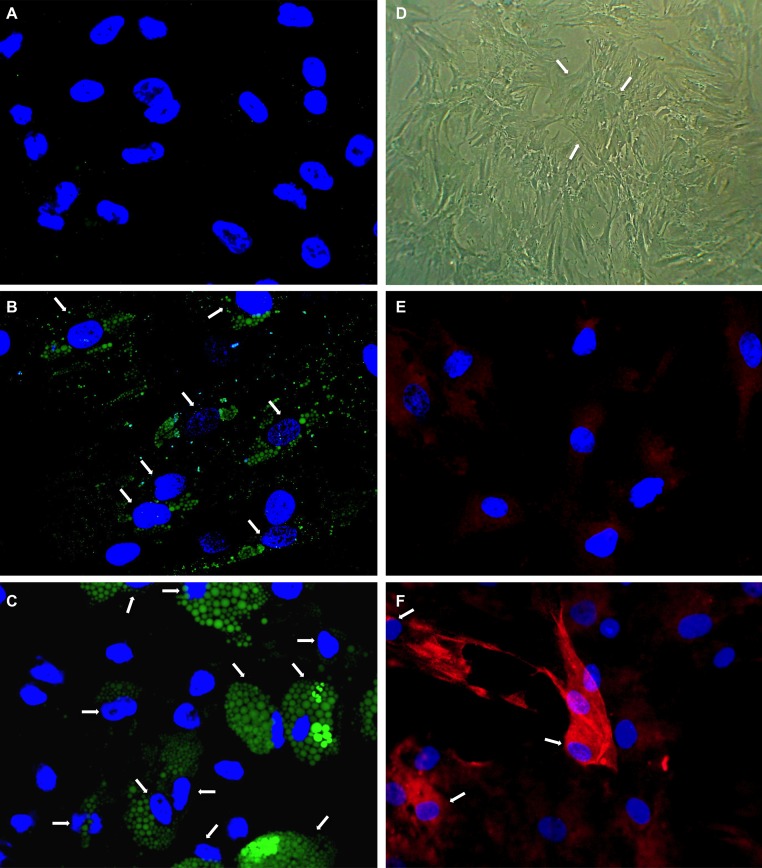
Analysis of adipogenic and osteogenic differentiation Human mesenchymal stem cells (hMSCs) were induced to differentiate towards adipocytes and osteoblasts. Adipogenic differentiation was determined using AdipoRed^TM^ assay. No fluorescence staining of lipid vacuoles was detectable before differentiation induction (**A**). After two cycles (**B**) and three cycles (**C**) of adipogenic induction fluorescent lipid vacuoles (green) were detectable increasing in number and size. Osteogenic differentiation was visible by cuboidal cell morphologie (**D**) and osteocalcin-detection in red fluorescence (**F**) using immune fluorescence after 6 days. Immune fluorescence revealed only weak osteocalcin-staining after 3 days of differentiation (**E**). Nuclei were counterstained with DAPI. Arrows point to differentiating cells.

In detail, adipogenic differentiation was performed with up to 3 cycles of adipogenic induction for 3 days and subsequent maintenance of the cells without induction medium for 1–2 days. One complete cycle of adipogenic induction lasted 4–5 days and three complete cycles of adipogenic induction 12–14 days. After one cycle of adipogenic induction, we found lipid vacuoles in 5% of cells indicating adipogenic differentiation. After 2 and after 3 cycles of adipogenic differentiation we detected large lipid vacuoles in 10–20% and 50% of cells, respectively, by employing AdipoRedAssay (Figure 2B, 2C). Number and size of lipid vacuoles increased from 2 to 3 cycles. After 6 days of osteogenic differentiation we found morphologic alteration i.e. cell shapes with a cubic like phenotype and increased cell bodies (Figure 2D). After 3 days of osteogenic differentiation we found a weak immune fluorescence staining of osteocalcin (Figure 2E) and after 6 days of osteogenic differentiation an intense osteocalcin staining in 10–20% of the cells (Figure 2F).

### arrayCGH analysis of mesenchymal stem cells and during differentiation

DNA from mesenchymal stem cells was isolated 4 days after initial seeding and prior to first passaging and differentiation. This DNA served as control for genomic alteration that occurred during the further cell culture including cell passaging. After one cycle of adipogenic induction and maintenance there was only a small number of cells (5%) with detectable lipid vacuoles. While this amount of differentiating cells after one cycle of adipogenic induction was not sufficient for arrayCGH-based detection of gene amplification, arrayCGH analysis was possible after the completion of two cycles of adipogenic induction with subsequent cell culture maintenance i.e. after 9 days, and after the completion of three cycles of induction also followed by culture maintenance i.e. after 14 days. For osteogenic differentiation cells were grown for 3 and 7 days in osteogenic differentiation induction medium. These time points were selected to analyze amplification events early during differentiation at day three and after the detection of calcification at day seven. Later time points were not investigated due to the impact of the increasing calcification on DNA quality.

As detailed in Materials and Methods arrayCGH was performed using Agilent-021529 SurePrint G3 Human CGH Microarray Kit and arrayCGH results were deposited in GEO under accession number GSE88947. After normalization we included a segmentation step using “runDNAcopy” from the packages snapCGH and DNAcopy (version 1.44.0) with alpha set to 0.01 and smooth region to 10. Merging was performed using “mergeStates” from (package snapCGH) with merge type set to 2 and default parameters. Log values above 0.2 were defined as amplified and log values below -0.2 as under-replicated chromosome regions. Chromosome regions with similar alteration in all samples were regarded as CNVs (present in Database of Genomic Variants available at UCSC genome Browser) and further eliminated. We identified 12 amplified and 17 under-replicated chromosome regions in undifferentiated hMSCs, 18 amplified and 18 under-replicated chromosome regions during adipogenic differentiation, and 19 amplified and 20 under-replicated chromosome regions during osteogenic differentiation. Results for amplified chromosome regions are summarized in Supplementary Table 1 and results for under-replicated chromosome regions in Supplementary Table 2.

### Fluorescence *in situ* hybridization of selected amplified chromosome regions

**Figure 3:**
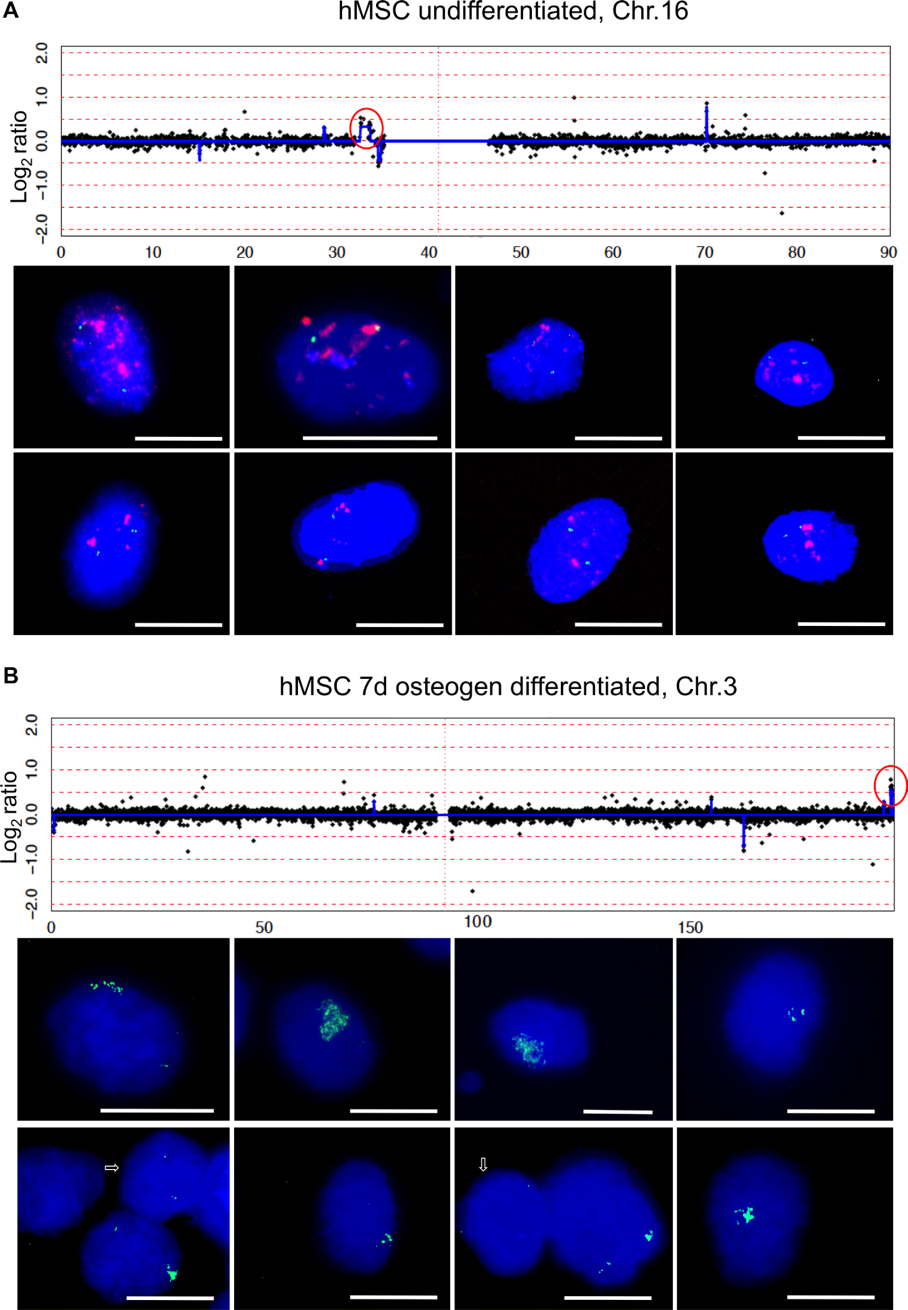
Amplification of chromosome region 16p11.2 and 3q29 ArrayCGH detected amplification of chromosome region 16p11.2 in undifferentiated hMSCs (**A**) and amplification of chromosome region 3q29 in hMSCs after 7 days differentiation towards osteoblasts (**B**). Log_2_ ratio plots were displayed with x-axis scale in Mb. Amplified chromosome regions that are circled in red were further investigated by fluorescence *in situ* hybridization. Examples of FISH results were shown for BAC RP11-53C21 (red) 16p11.2 and for BAC RP11-118F19 (green) 16q24.1. BAC RP11-53C21 reveals many and enlarged red fluorescence signals indicative of amplification whereas BAC Rp11-118F19 reveals only two green fluorescence signals indicative of normal diploid copy number (a). BAC RP11-728G2 (green) 3q29 reveals many green fluorescence signals with varying copy number as shown in eight representative nuclei with amplifications and arrows point to two nuclei with normal diploid copy number of BAC RP11-728G2. Nuclei were counterstained with DAPI. Size calibration bar = 5 μm.

Based on the arrayCGH results that revealed amplification within chromosome region 16p11.2 in undifferentiated mesenchymal stem cells (hMSCs) we employed FISH to confirm gene amplification using BAC probe RP11-53C21. BAC RP11-118F19 from chromosome 16q was used as control. Analyzing 100 nuclei from undifferentiated mesenchymal stem cells we found amplification of 16p11.2 in 15–20% of the cells indicated by large and frequently overlapping fluorescence signals of RP11-53C21. BAC RP11-118F19 showed two signals indicating a normal copy number for chromosome 16. Representative nuclei with the amplified chromosome 16p11.2 region are shown in Figure 3A. Next, we employed FISH to confirm the amplification that was detected by arrayCGH within chromosome region 3q29 of hMSCs that were differentiated for 7 days towards osteoblasts. Again, analyzing 100 nuclei we identified amplification in 5% of hMSC using BAC probe RP11-728G2 that maps within chromosome band 3q29 (Figure 3B).

### *CDK4* and *MDM2* amplification in human mesenchymal stem cells

**Figure 4:**
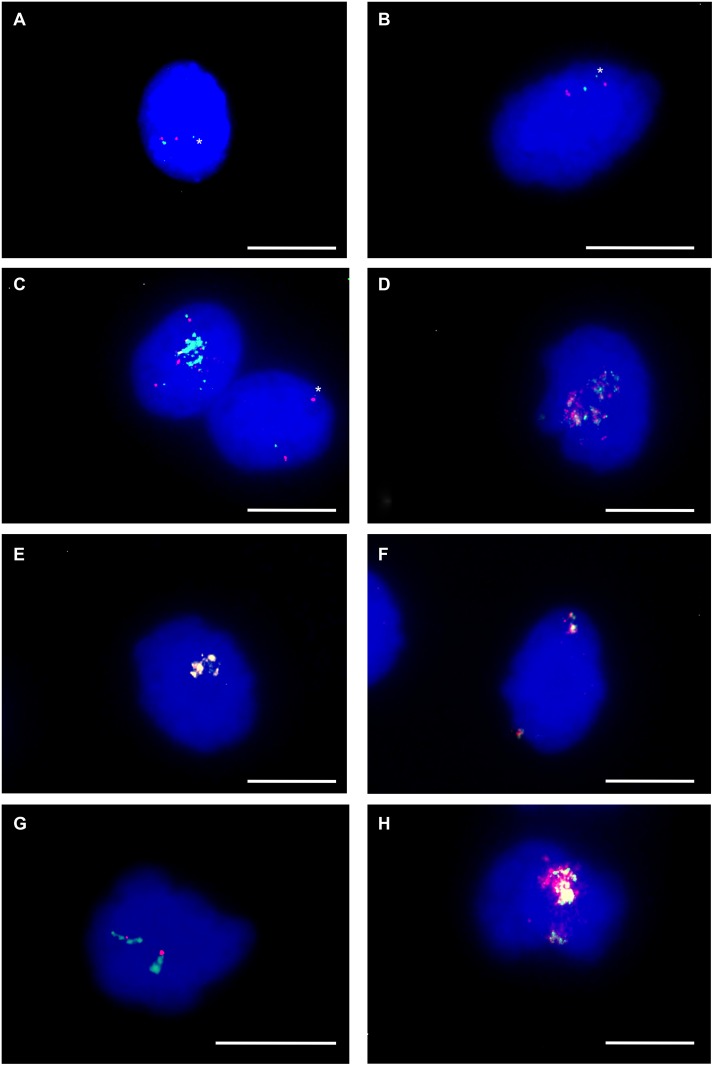
CDK4 and MDM2 amplification *CDK4* and *MDM2* amplification was analyzed using fluorescence *in situ* hybridization in undifferentiated, adipogenic-differentiated and osteogenic-differentiated hMSCs. In undifferentiated hMSCs FISH reveals two fluorescence signals for BAC RP11-611O2 (red) *MDM2* and two fluorescence signals for BAC RP11-571M6 (green) *CDK4* (**A**). FISH reveals cells with normal copy number (**B**, **C**) and with co-amplification (**C, D**) of *CDK4* and *MDM2* during first maintenance period of adipogenic differentiation (4 days). FISH reveals co-amplification of *MDM2* and *CDK4* (overlap of red and green signals) in hMSCs differentiated towards adipocytes for seven days (**E**, **F**). FISH reveals amplification of *CDK4* (green) with normal copy number of *MDM2* (red) (**G**) and co-amplification of *CDK4* and *MDM2* in hMSCs differentiated towards osteoblasts for seven days (**H**). Asterisks indicate weak single copy fluorescence signals of *CDK4*. Nuclei were counterstained with DAPI. Size calibration bar = 5 μm.

We used FISH for amplification analysis of *CDK4* and *MDM2* on single cell level. Previous investigations of neural stem cells and myoblasts during differentiation revealed overlap with amplifications found in tumors from the same lineage of cells. Specifically, *CDK4* and *MDM2* were frequently amplified in liposarcoma and osteosarcoma. As shown in Figure 4A FISH failed to detect gene amplification for *CDK4* at 12q14.1 and for *MDM2* at 12q15 in undifferentiated hMSC. For adipogenic differentiation we investigated 100 nuclei of mesenchymal stem cell after 4 days i.e. during first maintenance time and after 7 days i.e. after two cycles of adipogenic induction. After 4 days we detected co-amplification of *CDK4* and *MDM2* in 15% of the cells. The copy number of both *CDK4* and *MDM2* varied from cell to cell. An example of *CDK4* amplification higher than the *MDM2* amplification is shown in Figure 4C. Figure 4D shows the reverse situation with *MDM2* amplification higher than the *CDK4* amplification. The majority of cells showed a normal copy number of *CDK4* and *MDM2* as shown in Figure 4B and 4C. After 7 days of adipogenic differentiation i.e. after two cycles we found *CDK* and *MDM2* amplification in 20% of cells. Representative examples are given in Figure 4E and 4F. After 7 days of osteogenic differentiation, FISH also identified cells with co-amplifications of *CDK4* and *MDM2* (Figure 4H). In addition, cells that were differentiated towards osteoblasts for 7 days showed *CDK4* amplifications next to a normal copy number of *MDM2* (Figure 4G).

### Confirmation of gene amplification with qPCR

**Figure 5:**
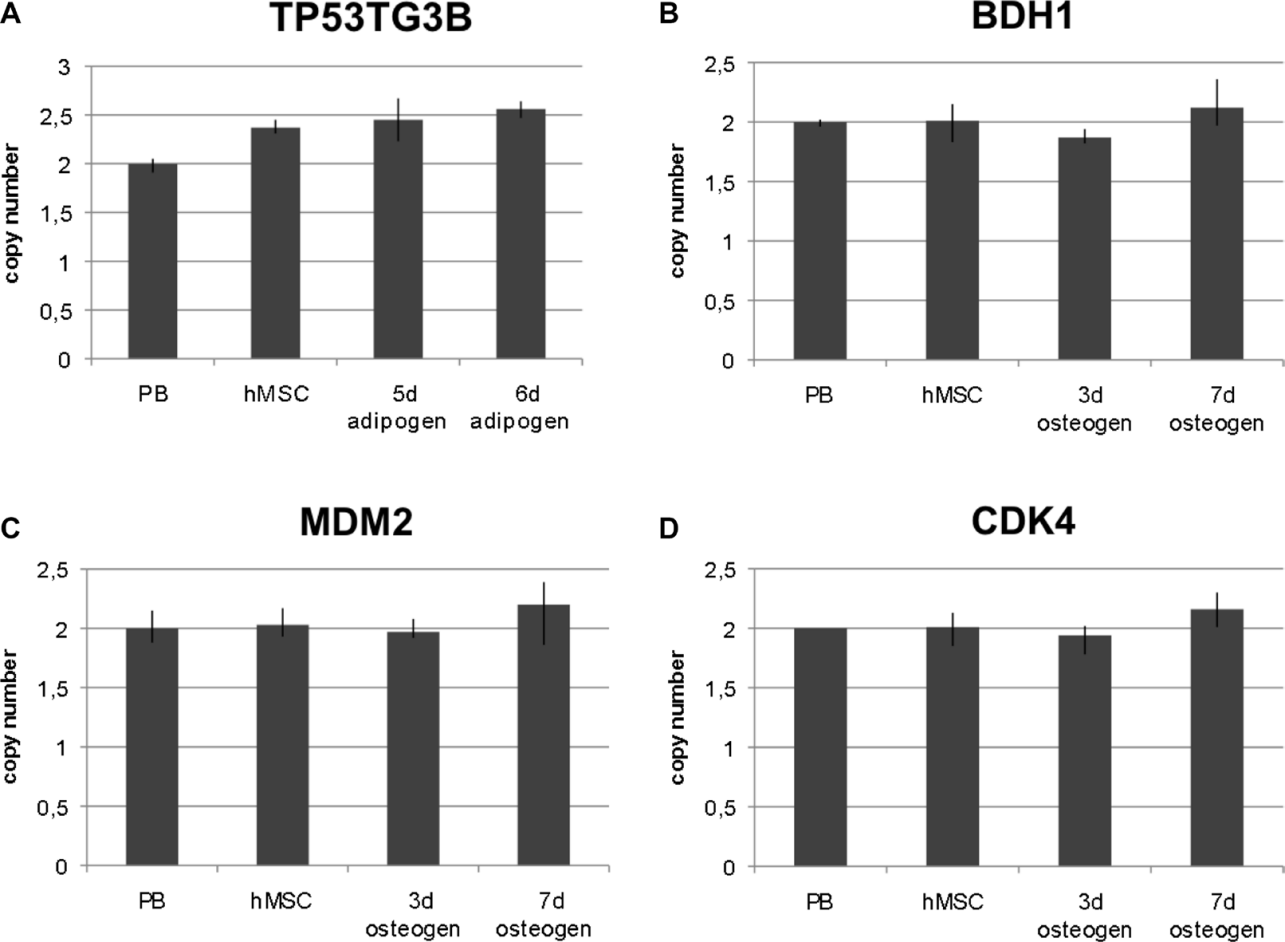
QPCR-analysis of selected amplified chromosome regions QPCR analysis was performed to further confirm gene amplification of four chromosome regions in a gene-specific approach. Amplification was analyzed in undifferentiated hMSCs, at two time points of early adipogenic differentiation, namely after first maintenance period and after one day induction period of the second cycle (5 days and 6 days,) and at two time points of osteogenic differentiation (3 days and 7 days). *TP53TG3B* represents chromosome region 16p11.2 (at 32.5 Mb), *BDH1* represents chromosome region 3q29 (at 197.2 Mb), *MDM2* represents chromosome region 12q15 (at 69.2 Mb) and *CDK4* represents chromosome region 12q14 (at 58.1 Mb). Amplification of *TP53TG3B* was detected in undifferentiated hMSCs and in adipogenic differentiated hMSCs (**A**). Amplification of *BDH1* was only detected in osteogenic differentiated hMSCs after 7 days (**B**). Amplification of *MDM2* and *CDK4* were detected in osteogenic differentiated hMSCs after 7 days (**C**, **D**). RNaseP was used as reference gene in TaqMan assays and DNA from normal lymphocytes (PB) served as standard for diploid copy number. Copy numbers are shown as mean from four technical replicates. Vertical lines indicate the range.

To confirm gene amplifications we used qPCR as an independent technique. The amplification was determined by qPCR analysis (TaqMan) in four replicates with the data analyzed by the software “copy caller“ (Applied Biosystems). Selection of amplified chromosome regions and of corresponding genes for qPCR was based on arrayCGH and FISH results. In detail, we determined copy numbers of the genes *TP53TG3B*, *BDH1, MDM2* and *CDK4* in undifferentiated hMSCs, in hMSCs differentiated towards adipocytes and in hMSCs differentiated towards osteoblasts. As for adipogenic differentiation, gene copy number was analyzed after 5 days i.e. a complete cycle of adipogenic induction and after 6 days i.e. one complete cycle followed by one day of the second cycle. As for osteogenic differentiation, gene copy number was analyzed after 3 and 7 days. *TP53TG3B* that maps at 16p11.2 was amplified in undifferentiated hMSCs and in adipogenic-differentiated hMSCs as shown in Figure 5A. *BDH1* that maps at 3q29 was not amplified in undifferentiated hMSC but in hMSCs that were differentiated for 7 days towards osteoblasts as shown in Figure 5B. Co-amplification of *MDM2* and *CDK4* was confirmed in the osteogenic differentiated hMSCs as shown in Figure 5C and 5D.

## DISCUSSION

We identified gene amplifications and under-replication in human mesenchymal stem cells and during their differentiation towards adipocytes and osteoblasts. Mesenchymal stem cells are also capable to differentiate towards chondrocytes, which could, however, not by analyzed by fluorescence *in situ* hybridization that required one dimensional tissue cultures. To investigate gene amplifications and under-replication we employed three independent techniques to compensate for the inherent limitations of each approach. Crucial for the analysis of amplifications by arrayCGH was the data processing and threshold setting. Standard data processing and threshold setting of log2 ratio above 0.8 for amplification and below –0.8 for deletion is suitable for homogeneous cell population and homogeneous tumor tissue but not for heterogeneous cell populations [10]. We applied a segmentation step and a low threshold for sensitive detection of gene amplifications and under-replications in heterogeneous cell populations. For example in hMSCs adipogenic differentiation induction led to 5% of cells with lipid vacuoles after one cycle of induction and to 10–20% of cells with lipid vacuoles after two cycles of induction. ArrayCGH was able to detect 14 amplified chromosome regions and 15 under-replicated chromosome regions after 2 cycles of induction. Both qPCR analysis and fluorescence *in situ* hybridization confirmed the arrayCGH results. However, amplifications found by single gene analysis were not necessarily identified by arrayCGH. For example, arrayCGH did not detect the copy number change on chromosome 12q14.1 and 12q15. These chromosome bands harbor the genes *CDK4* and *MDM2* that are amplified during adipogenic and osteogenic differentiation as revealed by FISH. In conclusion arrayCGH can discover amplified chromosome regions but does not necessarily identify short amplified sequences, which can be visualized by FISH. FISH is in turn not primarily suited to display the extension of amplified chromosome region. To compensate for the limitation of these techniques, we applied both arrayCGH and techniques suited for single gene analysis including FISH and qPCR.

Comparison of the amplification detected in human mesenchymal stem cells with results from our previous investigations on human neural stem and progenitor cells, we did not find major overlaps of the amplified chromosome regions. Out of 36 different amplified chromosome regions in undifferentiated or differentiated hMSC, only 4 chromosome regions were amplified in differentiated neural progenitor cells. We assume that gene amplification during differentiation appears to be a highly specific process. This assumption is further supported by two recent studies on gene amplification of human trophoblast with *ERBB2* amplification and on mouse trophoblast giant cells with five amplified regions [6, 7]. We did not detect those amplified chromosome regions in hMSCs. Amplification of chromosome region 17q12 including ERBB2 was, however, detected in 5-day differentiated neural progenitor cells [2]. Exceptions from this cell lineage specificity are genes *CDK4* and *MDM2* both localized on chromosome 12. *CDK4* was highly amplified in neural stem cells and neural progenitor cells both in an undifferentiated and a differentiated status, in myoblasts during differentiation and as shown in our present study in hMSCs during osteogenic differentiation. *MDM2* was highly amplified in myoblasts during differentiation and in neural stem cells during differentiation and again in hMSCs during osteogenic differentiation.

Previous investigations revealed that genes that were amplified in tumors of a specific cell origin were also frequently amplified in the corresponding line of stem cells during differentiation. *CDK4* and *MDM2* were amplified during differentiation of neural progenitor cells, in neural stem cells, and in glioblastoma [11, 12]. *CDK4* and *MDM2* were also amplified during differentiation of myoblasts towards myotubes, in leiomyosarcoma and rhabdomyosarcoma [13]. Consistent with these observations is the finding of CDK4 and *MDM2* amplifications not only in mesenchymal stem cells during differentiation towards osteoblasts and adipocytes but also in osteosarcoma and liposarcoma [7, 8].

Although gene amplification is known to occur during *D.melanogaster* development, human gene amplification was for decades primarily linked to tumor development and to multi-drug resistance. Recent studies show that amplification can have a physiological function during normal mammalian differentiation and it appears intriguing to hypothesis that the frequent amplifications in human tumor cells are adopted as mechanism from normal cells.

## MATERIALS AND METHODS

### Cell culture and differentiation

Human mesenchymal stem cells (hMSC) were obtained from Lonza (Walkersville Inc. USA) as primary cells frozen in passage 2 (P2). MSC from Lonza express CD29, CD44, CD105, CD166, CD90 and CD73 but do not express CD14, CD34 and CD45 and this is according to marker expression previously reported for bone marrow derived mesenchymal stem cells [14, 15]. Cells were passaged once, used in P3 for all experiments with a maximum culture time of 22 days from initial seeding to three complete cycles of adipogenic differentiation induction. HMSC were cultured until they become near confluent. Adipogenic differentiation was induced by 3 cycles of induction and maintenance. Each cycle consists of feeding with adipogenic induction medium for 3 days followed by culture in adipogenic maintenance medium for 1–2 days. Osteogenesis induction (osteogenic differentiation) was induced by osteogenesis medium for 7 days. Culture and media were according to Lonza instructions for use of Poietics^TM^ human mesenchymal stem cells.

### Immune fluorescence and adipored assay

HMSCs were cultivated on glass coverslips. Subsequently, cells were differentiation induced as described above. Osteogenic differentiation induced cells were methanol fixed and treated with 0,2% Tween-20 for 2 minutes. Coverslips were blocked with goat serum and incubated for 1 h with mouse antibody monoclonal to Osteocalcin (ab13418, Abcam). Detection was done with an Alexa-594 coupled secondary antibody against mouse.

Adipogenic differentiation induced cells were stained with AdipoRed Reagent (Lonza) according to manufacturers’ instructions.

### Fluorescence *in situ* hybridization

BAC clones were taken from the RP-11 (http://www.chori.org/bacpac/) libraries of the Welcome Trust Sanger Institute and available from SourceBioSciences, Germany [16]. BAC-DNA (1 μg) was either labeled with alexa 488-dUTP, with alexa 555-dUTP or with alexa 594-dUTP using the FISHTag DNA labeling Kit according to the manufacturer's instructions. Differentially labeled probe DNAs were precipitated in the presence of human Cot-1 DNA. Samples were resuspended in hybridization mix (50% formamide, 20×SSPE, 20% dextrane sulfate and 27% SDS).

Human mesenchymal stem cells and differentiating human mesenchymal stem cells were grown on glass slides and fixed in ice-cold methanol for 15 minutes. Slides were washed in PBS for 5 minutes and treated with 0, 02% Tween 20 for 5 minutes. Slides were washed again in PBS for 5 minutes. Slides were RNase treated for 1 h at 37°C and pepsin treated for 10 minutes. Postfixation was performed using 2, 5% formaldehyde/ 1×PBS for 10 minutes at room temperature. Slides were dehydrated by an ascending ethanol series (70%, 80%, 96%). Hybridization was performed by adding 12, 5 μl of the samples in hybridization mix to the slides. Posthybridization washes were as described previously [2].

Fluorescence images were captured with an Olympus AX70 microscope using CellSens software from Olympus.

### qPCR analysis

TaqMan Copy Number Assays for genes *BDH1* (Hs00871544_cn), *CDK4* (Hs00957586_cn), *MDM2* (Hs00181272_cn) and *TP53TG3B* (Hs04463315_cn) were performed following manufacturer's instructions. We used the RNase P TaqMan Copy number reference assay for relative quantitation of copy number of target genes. DNA from human normal blood lymphocytes (PB) was used as control standard for normal diploid copy number. TaqMan assays were run in four technical replicates and results were analyzed using StepOne™ Software v2.0 and CopyCaller™ software.

### Analysis of array-CGH data

ArrayCGH was performed using Agilent-021529 SurePrint G3 Human CGH Microarray Kit, 1×1M, design ID 21529 [hg19:GRCh37:Feb2009]. Genomic DNA was isolated from undifferentiated mesenchymal stem cells and from two time points of differentiation each for adipocyte and osteoblast differentiation. DNA from normal blood lymphocytes served as reference. ArrayCGH results were deposited in GEO under accession number GSE88947.

The analysis of the arrays was performed using R (https://www.R-project.org/ R version 3.2.3.), a language for statistical computing. The arrays were read in using “read.maimages” from the R Bioconductor package limma version 3.26.5 [17]. Positional information was added by a modified version of the method “readPositionalInfo” from the R Bioconductor package snapCGH (version 1.40.0) to be able to parse mitochondrial chromosome ID and to add a column “Position” containing the same information as the probe start column needed for further analysis steps. Since Cy3 was used as the reference the design attribute of the read in data was set appropriately. Background correction was performed applying function “backgroundCorrect” (package limma) with method “minimum”. The arrays were first normalized separately using “printtiploess” from “normalizeWithinArrays” and then quantile normalized using “normalizeBetweenArrays” (package limma). As next, the normalized data of chromosomes 1 to 22 was further processed using “processCGH” (package snapCGH). In order to minimize noise, for each chromosome the probes were assigned to intervals of 25kb and their value set to the interval mean. The segmentation was done using “runDNAcopy” from the packages snapCGH and DNAcopy (version 1.44.0) with alpha set to 0.01 and smooth region to 10 and merging was performed using “mergeStates” from (package snapCGH) with merge type set to 2 and default parameters. Finally, for each array a state table was created: The probes were divided into intervals based on the chromosome and the merged segmentation state and their start and end positions, and the predicted and average observed values were saved.

## SUPPLEMENTARY MATERIALS FIGURES AND TABLES







## Abbreviations

array-CGH: array comparative genomic hybridization
BDH1: D-Beta-Hydroxybutyrate Dehydrogenase 1
CDK4: cyclin-dependent kinase 4
CNV: copy number variation
FISH: fluorescence <italic>in situ </italic>hybridization
hMSC: human mesenchymal stem cell
MDM2: mouse double minute 2
PB: peripheral blood
PRSS1: Serine Protease 1
qPCR: quantitative polymerase chain reaction
TP53TG3B: TP53 target 3B
