# Supplementary material for: Gene amplification in mesenchymal stem cells and during differentiation towards adipocytes or osteoblasts
Source: Oncotarget. 2017 Dec 1;9(2):1803–12. doi: 10.18632/oncotarget.22804 (PMC5788600; doi:10.18632/oncotarget.22804)
Supplement: Supplementary file 1 [file oncotarget-09-1803-s001.pdf]

Gene amplification in mesenchymal stem cells and during differentiation towards adipocytes or osteoblasts

SUPPLEMENTARY MATERIALS

Supplementary Table 1: Overview on amplified chromosome region. See_Supplementary_Table 1

Supplementary Table 2: Overview on under-replicated chromosome regions. See_Supplementary_Table 2
