# Supplementary material for: Gene amplification in mesenchymal stem cells and during differentiation towards adipocytes or osteoblasts
Source: Oncotarget. 2017 Dec 1;9(2):1803–12. doi: 10.18632/oncotarget.22804 (PMC5788600; doi:10.18632/oncotarget.22804)
Supplement: Supplementary file 2 [file oncotarget-09-1803-s002.docx]

**Supplementary Table 1: Overview on amplified chromosome region**

|  | hMSC | | | 2 cycles adipogenic differentiation | | | 3 cycles adipogenic differentiation | | | 3-day osteogenic differentiation | | | 7-day osteogenic differentiation | | |
| --- | --- | --- | --- | --- | --- | --- | --- | --- | --- | --- | --- | --- | --- | --- | --- |
| Chromosome | mean log_2_ ratio value per segment | Start* | End* | mean log_2_ ratio value per segment | Start* | End* | mean log_2_ ratio value per segment | Start* | End* | mean log_2_ ratio value per segment | Start* | End* | mean log_2_ ratio value per segment | Start* | End* |
| 1 | 0.44 | 17,207,777 | 17,254,504 | 0.41 | 17,207,777 | 17,254,504 | 0.42 | 17,207,777 | 17,254,504 |  |  |  |  |  |  |
| 1 | 0.44 | 169,214,647 | 169,263,915 | 0.41 | 169,214,647 | 169,263,915 |  |  |  |  |  |  |  |  |  |
| 2 | 0.49 | 97,815,934 | 97,823,863 |  |  |  |  |  |  |  |  |  |  |  |  |
| 3 | 0.24 | 75,762,756 | 75,793,273 |  |  |  |  |  |  |  |  |  | 0.28 | 75,762,756 | 75,793,273 |
| 3 |  |  |  |  |  |  | 0.34 | 89,387,958 | 89,433,426 |  |  |  |  |  |  |
| 3 |  |  |  |  |  |  |  |  |  |  |  |  | 0.28 | 154,949,239 | 154,979,106 |
| 3 |  |  |  |  |  |  |  |  |  |  |  |  | 0.28 | 195,339,139 | 195,486,455 |
| 3 |  |  |  |  |  |  |  |  |  | 0.49 | 197,038,043 | 197,485,688 | 0.54 | 197,038,043 | 197,485,688 |
| 4 |  |  |  |  |  |  |  |  |  | 0.44 | 0,302,397 | 0,449,774 | 0.40 | 0,302,397 | 0,449,774 |
| 4 |  |  |  |  |  |  |  |  |  | 0.44 | 70,096,386 | 70,259,782 |  |  |  |
| 4 |  |  |  |  |  |  |  |  |  | 0.44 | 126,154,192 | 126,198,491 |  |  |  |
| 5 | 0.65 | 0,776,954 | 0,820,424 | 0.65 | 0,776,954 | 0,820,424 | 0.60 | 0,759,169 | 0,820,424 |  |  |  |  |  |  |
| 5 | 0.65 | 35,502,637 | 35,574,804 | 0.65 | 35,502,637 | 35,545,651 | 0.60 | 35,502,637 | 35,545,651 |  |  |  |  |  |  |
| 5 |  |  |  |  |  |  |  |  |  |  |  |  | 0.39 | 70,308,101 | 70,369,959 |
| 6 | 0.31 | 0,255,350 | 0,378,956 | 0.32 | 0,255,350 | 0,378,956 | 0.30 | 0,255,350 | 0,378,956 |  |  |  |  |  |  |
| 6 |  |  |  |  |  |  | 0.68 | 26,741,743 | 26,760,484 |  |  |  |  |  |  |
| 6 |  |  |  | 0.32 | 67,011,952 | 67,052,417 | 0.30 | 67,011,952 | 67,052,417 |  |  |  | 0.45 | 67,011,952 | 67,052,417 |
| 6 |  |  |  |  |  |  |  |  |  | 0.46 | 165,705,451 | 165,754,013 | 0.45 | 165,705,451 | 165,754,013 |
| 7 |  |  |  |  |  |  | 0.25 | 38,302,628 | 38,376,560 |  |  |  | 0.25 | 38,302,628 | 38,376,560 |
| 7 |  |  |  | 0.26 | 142,328,008 | 142,500,331 | 0.25 | 142,328,008 | 142,500,331 |  |  |  | 0.25 | 142,328,008 | 142,500,331 |
| 7 |  |  |  |  |  |  |  |  |  |  |  |  | 0.25 | 143,203,385 | 143,519,863 |
| 8 |  |  |  |  |  |  | 0.41 | 11,906,207 | 12,240,412 |  |  |  |  |  |  |
| 11 |  |  |  |  |  |  |  |  |  | 0.47 | 49,726,476 | 49,765,437 |  |  |  |
| 11 |  |  |  | 0.31 | 89,468,017 | 89,656,971 | 0.38 | 89,468,017 | 89,549,648 |  |  |  |  |  |  |
| 12 |  |  |  |  |  |  |  |  |  | 0.54 | 34,756,150 | 37,958,199 |  |  |  |
| 14 |  |  |  |  |  |  |  |  |  | 0.36 | 19,376,762 | 19,728,700 | 0.34 | 19,376,762 | 19,728,700 |
| 14 | 0.69 | 19,728,641 | 20,424,635 | 0.61 | 19,376,762 | 20,424,635 | 0.64 | 19,376,762 | 20,424,635 |  |  |  |  |  |  |
| 15 |  |  |  |  |  |  |  |  |  | 0.65 | 34,727,647 | 34,806,953 | 0.40 | 34,727,647 | 34,806,953 |
| 16 | 0.32 | 28,569,216 | 28,632,039 |  |  |  |  |  |  |  |  |  |  |  |  |
| 16 | 0.32 | 32,573,808 | 33,630,726 | 0.33 | 32,573,808 | 33,630,726 | 0.35 | 32,573,808 | 33,630,726 |  |  |  |  |  |  |
| 16 |  |  |  | 0.33 | 74,372,285 | 74,407,341 | 0.35 | 74,372,285 | 74,407,341 | 0.62 | 74,372,285 | 74,407,341 | 0.69 | 74,372,285 | 74,407,341 |
| 18 |  |  |  |  |  |  |  |  |  | 0.46 | 41,173,917 | 41,206,594 | 0.41 | 41,173,917 | 41,206,594 |
| 19 |  |  |  | 0.41 | 20,574,452 | 20,607,913 |  |  |  |  |  |  |  |  |  |
| 21 | 0.25 | 11,035,537 | 11,145,601 |  |  |  |  |  |  |  |  |  |  |  |  |
| 21 |  |  |  | 0.56 | 14,420,615 | 14,640,451 |  |  |  |  |  |  |  |  |  |
| 22 | 0.60 | 24,337,608 | 24,408,525 | 0.75 | 24,337,608 | 24,408,525 | 0.71 | 24,337,608 | 24,408,525 |  |  |  |  |  |  |

*Start and end point of amplified chromosome regions were according to GRCh37/HG19.
