# Supplementary material for: Gene amplification in mesenchymal stem cells and during differentiation towards adipocytes or osteoblasts
Source: Oncotarget. 2017 Dec 1;9(2):1803–12. doi: 10.18632/oncotarget.22804 (PMC5788600; doi:10.18632/oncotarget.22804)
Supplement: Supplementary file 3 [file oncotarget-09-1803-s003.docx]

**Supplementary Table 2: Overview on under-replicated chromosome regions**

|  | hMSC | | | 2 cycles adipogenic differentiation | | | 3 cycles adipogenic differentiation | | | 3-day osteogenic differentiation | | | 7-day osteogenic differentiation | | |
| --- | --- | --- | --- | --- | --- | --- | --- | --- | --- | --- | --- | --- | --- | --- | --- |
| Chromosome | mean log_2_ ratio value per segment | Start* | End* | mean log_2_ ratio value per segment | Start* | End* | mean log_2_ ratio value per segment | Start* | End* | mean log_2_ ratio value per segment | Start* | End* | mean log_2_ ratio value per segment | Start* | End* |
| 1 | -0.50 | 17,088,414 | 17,091,284 | -0.57 | 17,088,414 | 17,091,284 |  |  |  |  |  |  |  |  |  |
| 1 | -0.50 | 248,727,929 | 248,811,841 | -0.57 | 248,727,929 | 248,811,841 | -0.57 | 248,727,929 | 248,811,841 |  |  |  |  |  |  |
| 2 | -0.84 | 13,846,313 | 13,961,651 | -0.77 | 13,846,313 | 13,961,651 | -0.86 | 13,846,313 | 13,961,651 |  |  |  |  |  |  |
| 2 |  |  |  |  |  |  |  |  |  |  |  |  | -0.65 | 34,697,718 | 34,738,236 |
| 2 | -0.46 | 89,475,372 | 90,265,119 | -0.37 | 89,475,372 | 91,815,738 | -0.45 | 89,427,365 | 91,766,271 |  |  |  |  |  |  |
| 2 | -0.46 | 97,857,471 | 97,877,426 |  |  |  |  |  |  |  |  |  |  |  |  |
| 2 | -0.46 | 132,195,561 | 132,266,573 |  |  |  |  |  |  |  |  |  | -0.65 | 132,195,561 | 132,266,573 |
| 2 |  |  |  |  |  |  |  |  |  |  |  |  | -1.59 | 146,846,075 | 146,891,752 |
| 2 | -0.46 | 195,295,724 | 195,334,338 |  |  |  |  |  |  |  |  |  |  |  |  |
| 3 |  |  |  |  |  |  |  |  |  |  |  |  | -0.38 | 0,614,224 | 0,660,813 |
| 3 |  |  |  | -0.29 | 46,78,943 | 46,854,495 |  |  |  |  |  |  |  |  |  |
| 3 |  |  |  |  |  |  | -0.29 | 162,689,567 | 162,785,899 | -0.61 | 162,514,534 | 162,632,782 | -0.70 | 162,514,534 | 162,632,782 |
| 5 |  |  |  |  |  |  |  |  |  | -0.28 | 0,723,194 | 0,777,000 | -0.33 | 0,723,194 | 0,777,000 |
| 6 |  |  |  |  |  |  |  |  |  | -0.46 | 32,430,137 | 32,459,543 |  |  |  |
| 8 | -0.41 | 7,239,491 | 7,671,790 | -0.28 | 7,169,490 | 7,786,708 |  |  |  | -0.41 | 6,939,251 | 7,786,708 | -0.43 | 7,169,490 | 7,786,708 |
| 8 | -0.68 | 39,237,438 | 39,386,158 | -0.67 | 39,237,438 | 39,386,158 | -0.63 | 39,237,438 | 39,386,158 |  |  |  |  |  |  |
| 9 | -0.47 | 43,505,843 | 43,590,139 | -0.30 | 38,768,232 | 43,590,139 | -0.44 | 43,505,843 | 43,590,139 | -0.27 | 39,140,211 | 45,469,390 |  |  |  |
| 9 |  |  |  | -0.30 | 94,213,623 | 94,277,645 |  |  |  |  |  |  |  |  |  |
| 11 | -0.52 | 56,322,542 | 56,363,931 |  |  |  | -0.62 | 56,322,542 | 56,363,931 |  |  |  |  |  |  |
| 12 | -0.39 | 9,639,866 | 9,713,425 |  |  |  | -0.35 | 9,624,800 | 9,713,425 |  |  |  | -0.31 | 9,624,800 | 9,737,689 |
| 12 | -0.70 | 10,565,896 | 10,60,733 | -0.70 | 10,565,896 | 10,607,330 |  |  |  |  |  |  |  |  |  |
| 13 |  |  |  |  |  |  |  |  |  | -0.47 | 44,072,308 | 44,492,727 | -0.44 | 44,072,308 | 44,492,727 |
| 13 | -2.35 | 57,748,283 | 57,795,091 | -2.11 | 57,748,283 | 57,795,091 |  |  |  |  |  |  |  |  |  |
| 13 | -0.31 | 76,602,643 | 76,640,824 |  |  |  |  |  |  |  |  |  |  |  |  |
| 15 | -0.61 | 43,916,956 | 43,936,347 | -0.56 | 43,916,956 | 43,936,347 | -0.44 | 43,916,956 | 43,936,347 |  |  |  |  |  |  |
| 16 |  |  |  |  |  |  |  |  |  | -0.32 | 22,633,888 | 22,718,338 | -0.35 | 22,633,888 | 22,690,767 |
| 16 |  |  |  |  |  |  |  |  |  | -0.32 | 32,471,625 | 33,939,194 | -0.35 | 32,471,625 | 33,939,194 |
| 16 |  |  |  |  |  |  |  |  |  | -0.32 | 70,048,808 | 70,135,644 |  |  |  |
| 17 |  |  |  |  |  |  |  |  |  | -0.57 | 4,349,475 | 4,472,372 | -0.49 | 4,349,475 | 4,472,372 |
| 17 |  |  |  |  |  |  |  |  |  |  |  |  | -0.49 | 50,774,416 | 50,870,900 |
| 18 |  |  |  |  |  |  |  |  |  |  |  |  | -0.32 | 5,195,011 | 5,258,611 |
| 18 |  |  |  | -0.36 | 22,302,928 | 22,336,879 |  |  |  |  |  |  | -0.32 | 22,302,928 | 22,336,879 |
| 19 | -0.64 | 41,355,019 | 41,371,576 | -0.66 | 41,355,019 | 41,371,576 |  |  |  |  |  |  |  |  |  |
| 21 |  |  |  | -0.34 | 15,034,247 | 15,070,283 |  |  |  |  |  |  |  |  |  |
| 22 |  |  |  |  |  |  |  |  |  | -0.35 | 24,337,608 | 24,408,525 | -0.62 | 24,337,608 | 24,408,525 |
| 22 |  |  |  |  |  |  |  |  |  | -0.35 | 42,886,734 | 42,957,173 | -0.25 | 42,886,734 | 42,957,173 |

*Start and end point of under-replicated chromosome regions were according to GRCh37/HG19.
